# Supplementary material for: TastepepAI: An artificial intelligence platform for taste peptide de novo design
Source: PLoS Comput Biol. 2025 Oct 16;21(10):e1013602. doi: 10.1371/journal.pcbi.1013602 (PMC12543283; doi:10.1371/journal.pcbi.1013602)
Supplement: S1 File — (ZIP) [file pcbi.1013602.s006.zip › Supplementary Data 2/TaPep47 HPLC.doc]

**HPLC REPORT**

| Sample: | Fcl-NJP93350 TaPep47 IP-7 | | | Analyzed date: | 2024-9-23 |
| --- | --- | --- | --- | --- | --- |
| Analyst: | LY Reconstitution: 1mg/0.5ml H_2_O | | | | |
| Lot. No.: | P240913-MX1172281 | | | | |
| Column: | 250*4.6mm,ChromCore 120 C18 5um | | | | |
| Solvent A | A: 0.1% Trifluoroacetic Acid in 100% Acetonitrile | | | | |
| Solvent B | B: 0.1% Trifluoroacetic Acid in 100% Water | | | | |
| Gradient: |  | A | B |  | |
|  | 0.0min | 16% | 84% |  | |
|  | 25.0min | 41% | 59% |  | |
|  | 25.1min | 100% | 0% |  | |
|  | 30.0min | Stop | |  | |
| Volume: | 10μl | | |  | |
| Wavelength: | 220nm | | |  | |
| Flow rate: | 1.0ml/min | | |  | |
| ───────────────────────────────────────────────────  Peak No Ret Time Height Area Conc.  ───────────────────────────────────────────────────  1 10.600 15814.182 20526.688 0.2045  2 10.730 668081.625 9951563.000 99.1241  3 10.730 24263.918 66132.820 0.6587  4 21.112 164.078 1278.547 0.0127  ───────────────────────────────────────────────────  Total 100.00 | | | | | |
